# Proteomic characterization of serine hydrolase activity and composition in normal urine

**DOI:** 10.1186/1559-0275-10-17

**Published:** 2013-11-15

**Authors:** Mario Navarrete, Julie Ho, Oleg Krokhin, Peyman Ezzati, Claudio Rigatto, Martina Reslerova, David N Rush, Peter Nickerson, John A Wilkins

**Affiliations:** 1 Manitoba Centre for Proteomics & Systems Biology, 799 John Buhler Research Centre, 715 Mc Dermot Avenue, Winnipeg, Manitoba R3A 1R9, Canada; 2 Section of Nephrology, GE 421C Health Sciences Centre, University of Manitoba, 820 Sherbrook Street, Winnipeg, Manitoba R3A 1R9, Canada; 3 Section of Nephrology, Renal Health Program - Seven Oaks General Hospital/SBGH, University of Manitoba, 2PD02-2300 McPhillips Street, Winnipeg, Manitoba R2V 3M3, Canada; 4 Section of Nephrology, St. Boniface General Hospital, University of Manitoba, 409 Tache Avenue, Winnipeg, Manitoba R2H 2A6, Canada; 5 Organ & Tissues Office, Canadian Blood Services Building, Room 312-777 William Avenue, Winnipeg, Manitoba R3E 3P4, Canada

**Keywords:** Activity-based protein profiling, Catabolomics, Fluorophosphonate probe, Mass spectrometry

## Abstract

**Background:**

Serine hydrolases constitute a large enzyme family involved in a diversity of proteolytic and metabolic processes which are essential for many aspects of normal physiology. The roles of serine hydrolases in renal function are largely unknown and monitoring their activity may provide important insights into renal physiology. The goal of this study was to profile urinary serine hydrolases with activity-based protein profiling (ABPP) and to perform an in-depth compositional analysis.

**Methods:**

Eighteen healthy individuals provided random, mid-stream urine samples. ABPP was performed by reacting urines (n = 18) with a rhodamine-tagged fluorophosphonate probe and visualizing on SDS-PAGE. Active serine hydrolases were isolated with affinity purification and identified on MS-MS. Enzyme activity was confirmed with substrate specific assays. A complementary 2D LC/MS-MS analysis was performed to evaluate the composition of serine hydrolases in urine.

**Results:**

Enzyme activity was closely, but not exclusively, correlated with protein quantity. Affinity purification and MS/MS identified 13 active serine hydrolases. The epithelial sodium channel (ENaC) and calcium channel (TRPV5) regulators, tissue kallikrein and plasmin were identified in active forms, suggesting a potential role in regulating sodium and calcium reabsorption in a healthy human model. Complement C1r subcomponent-like protein, mannan binding lectin serine protease 2 and myeloblastin (proteinase 3) were also identified in active forms. The in-depth compositional analysis identified 62 serine hydrolases in urine independent of activity state.

**Conclusions:**

This study identified luminal regulators of electrolyte homeostasis in an active state in the urine, which suggests tissue kallikrein and plasmin may be functionally relevant in healthy individuals. Additional serine hydrolases were identified in an active form that may contribute to regulating innate immunity of the urinary tract. Finally, the optimized ABPP technique in urine demonstrates its feasibility, reproducibility and potential applicability to profiling urinary enzyme activity in different renal physiological and pathophysiological conditions.

## Background

Detailed knowledge of the composition and activities of urine proteins could provide novel insights into normal renal physiology. Although proteomic studies have identified a large number of urinary proteins [1-4] their functional relevance remains largely unknown. Furthermore, many proteomic studies do not account for post-translational modifications which may have a significant impact on protein function. Many proteins are enzymes that are maintained in a latent state until their activity is required. This allows for rapid host responses, without the time lag required for transcription and translation. Thus, there can be marked changes in functional states in the absence of significant alteration in concentration. These functional changes in activity are undetectable with methods that simply quantify transcript or protein levels, but are important for characterizing the dynamic physiological status of the host.

**Figure 1:**
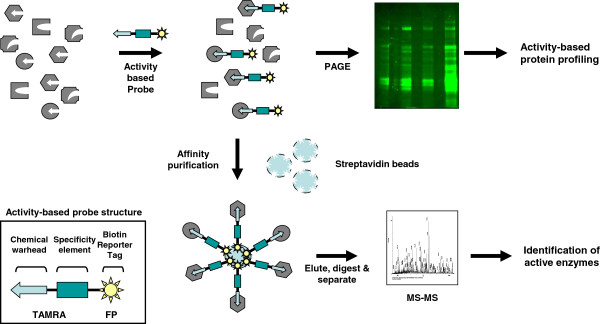
Overview of activity-based protein profiling.

Activity-based protein profiling (ABPP) is a novel approach to assess the functional status of selected enzymes in the proteome [5]. ABPP is based on the use of tagged probes that selectively react with the active sites of a given enzyme or family of enzymes [5,6]. Activity-based probes consist of a reactive group that targets the active residue of the enzyme, a short linker and reporter tag. The central premise of ABPP is that accessibility of substrate to the active site of an enzyme is an indicator of enzyme activation. Because the underlying molecular mechanisms of catalysis by members of an enzyme family are often identical [7] it is possible to develop a single probe to detect the active forms of members from a given family [8,9]. Members of the serine hydrolase family share a serine centric charge relay system in their catalytic site and this common feature can be exploited with an activity-based probe to selectively label active serine hydrolases. Furthermore, probe-labeled enzymes can be affinity-purified through their tag and identified by mass spectrometry to determine the specific active enzymes within a biological sample (Figure 1).

The serine hydrolase family is one of the largest enzyme classes in humans and constitutes ~1% of predicted protein products from the eukaryotic genome. Serine hydrolases consist of greater than 100 serine proteases and approximately 110 esterases, lipases, peptidases and amidases [10]. While some members are well-characterized (e.g. trypsin, elastase, thrombin, acetylcholinesterase), many have yet to be described [10]. Indeed the role for ~50% of the non-serine proteases remains undetermined [10], and very little is known about the presence and role of serine hydrolases in the urine of healthy individuals. Therefore the objective of this study was to evaluate the activity and composition of serine hydrolases in normal urine.

Thirteen serine hydrolases were identified in an active form in normal urine that may reflect regulation of renal electrolyte homeostasis and innate immunity of the urinary tract. The in-depth compositional analysis identified 62 serine hydrolases in normal urine independent of activity state. The ABPP technique that we optimized in urine is a powerful approach for functional proteomic screening by profiling and identification of enzymes in an active state.

## Results

### Demonstration of serine hydrolase activity in normal urine

**Figure 2:**
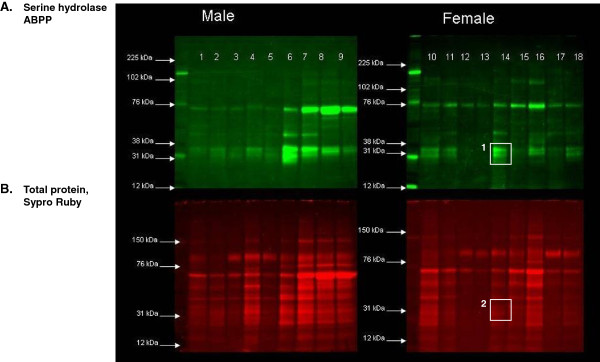
**Activity based protein profiling demonstrates consistent patterns of serine hydrolase activity in normal male (n = 9) and female urine (n = 9).(A)** Activity-based protein profiling (ABPP, green). **(B)** Total protein (Sypro Ruby, red). Although serine hydrolase activity tended to correlate with total protein, activity was sometimes independent of total protein. Example: Female 14, activity and protein content highlighted in panel **A** and **B**, respectively.

Initial studies were undertaken to determine if there was evidence of serine hydrolase activity in normal urine. Urines were reacted with a fluorophosphonate probe tetramethylrhodamine (FP-TAMRA) which detects a broad range of serine hydrolase activities independent of the specific reactions that these enzymes catalyze. Equal volumes of random mid-stream urines from 18 healthy donors were individually reacted with FP-TAMRA probe under standardized optimal conditions. The proteins were separated by SDS-PAGE and the fluorescently labeled active enzymes were visualized in gel (Figure 2).

Seven to ten distinct fluorescent bands of varying intensity were observed in each sample with some evidence of numerous weaker fluorescing species (Figure 2). There were several common features in the majority of the samples with bands in the 31 kDa and 75 kDa regions of all samples. Several weaker staining bands in the 102–110 kDa and 40–50 kDa regions were also observed in a subset of the samples. There did not appear to be any gender-based differences in activity patterns. The degree of FP-TAMRA staining demonstrated frequent but not exclusive, correlation with total protein composition as detected by Sypro Ruby, which suggests that enzyme quantity is closely but not exclusively correlated with enzyme activity. Finally, the amount of FP-TAMRA labeled bands was significantly less than the total protein composition as detected by Sypro Ruby, suggesting that the enzymes detected by the activity probe represent a minor component of the total urinary protein pool (Figure 2).

**Figure 3:**
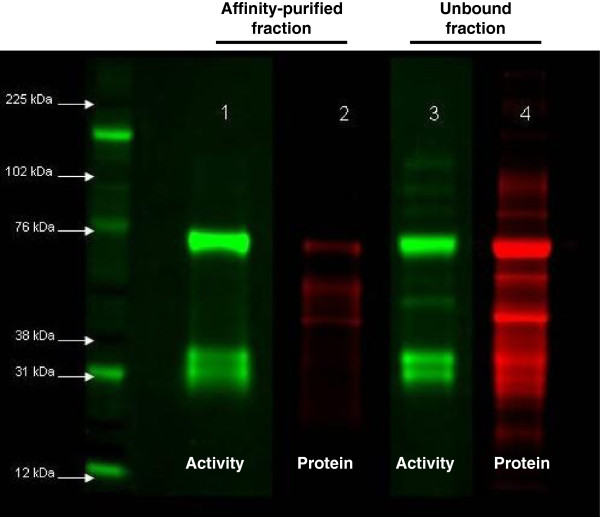
**Immunoprecipitation of active serine hydrolases from normal urine.** Affinity purification demonstrated enrichment of labeled bands (ABPP, green) relative to total protein content (Sypro Ruby, red). Active bands were cut from Lane 1 and in-gel digestion performed for enzyme identification with LC-MS/MS.

The FP-TAMRA labeled proteins in normal urine were affinity purified with an anti-TAMRA antibody [11] and analyzed by mass spectrometry as an approach to identifying the specific active serine hydrolases in normal urine. The anti-TAMRA purified material was highly enriched in labeled proteins but markedly reduced in protein content relative to the starting material (Figure 3). The gel regions containing the FP-TAMRA labeled bands were cut and processed for MS/MS. In a complementary approach, affinity-purified FP-TAMRA labeled proteins were digested in-solution and identified by MS/MS. The affinity purification with anti-TAMRA antibody was specific as demonstrated by the lack of enrichment of TAMRA labeled proteins by an irrelevant antibody to HIV gp120 (Additional file 1).

Thirteen active serine hydrolases were identified by MS/MS following the in-gel or in-solution digestion. The protein identifications were based on high confidence scores ranging from a log_10_ (-3.2) to log_10_ (-128.6) using the Global Proteome Machine (http://www.thegpm.org). A comparison of the predicted molecular weights of these proteins based on amino acid sequence and their molecular weights as estimated by their positions on the SDS PAGE gels were consistent with several of the labeled species observed in Figure 2. This provided further support for the assigned identities of the enzymes isolated by in-solution digestion.

**Figure 4:**
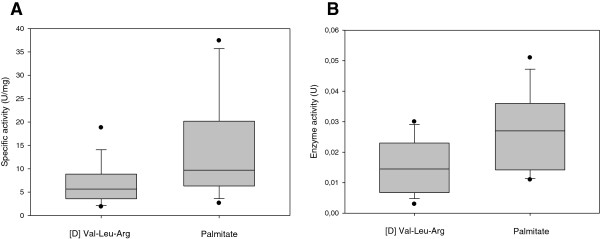
**Quantitative enzyme activity assays demonstrating the range of normal urinary enzyme activity, both uncorrected (A) and corrected for urinary protein (B).** Bile-salt activated lipase activity was demonstrated with the substrate palmitate, which releases 4-nitrophenol, measured in μmol/mL/min [U]. Tissue kallikrein and urokinase activity are demonstrated with the substrate [D] Val-Leu-Arg which releases paranitronilide, measured in μmol/mL/min [U].

The presence of serine hydrolase activity in normal urines was directly assessed by incubating the urines (n = 18) with one specific substrate and monitoring for the generation of a reaction product. Evidence of carboxyl ester lipase (bile salt-activated lipase) activity was demonstrated using 4-nitrophenol palmitate as substrate (Figure 4) [12]. The presence of kallikrein-1 (tissue kallikrein) and urokinase activity was demonstrated using the [D] Val-Leu-Arg-paranitronilide substrate (Figure 4) [13]. Collectively, these results confirmed the presence of several serine hydrolase activities in normal urine.

### Serine hydrolase composition of normal urine

Although the previous results identified the presence of active urinary serine hydrolases it was apparent that not all of the FP-TAMRA labeled species were isolated by affinity purification (Figure 3). Therefore, a 2D LC-MS/MS analysis of pooled healthy urine samples (n = 4) was undertaken as an alternate approach to determining the serine hydrolase composition of normal urine. The goal was to characterize the overall normal urinary serine hydrolase population; therefore pooled urines were utilized for biological averaging in lieu of individual biological replicates. A total of 1846 urinary proteins were identified and these lists were annotated to identify all the potential serine hydrolases. A total of 62 serine hydrolases were identified with high confidence GPM log_10_ (expectation) scores of less than -3 (Table 1).

**Table 1:** Serine hydrolases detected in normal urine (n=62)

**Serine hydrolase**	**Uniprot number**	**Log(e)**^**§**^
Acylamino-acid-releasing enzyme	P13798	-109.3
Alpha/beta hydrolase domain-containing protein 14B	Q96IU4	-81
Apolipoprotein(a)	P08519	-164.5
Azurocidin	P20160	-5
Bile salt-activated lipase*	P19835	-352.6
Cholinesterase	P06276	-10.7
Chymotrypsin-like protease CTRL-1	P40313	-7.4
Coagulation factor VII	P08709	-26.3
Coagulation factor IX	P00740	-33.4
Coagulation factor XI	P03951	-80.5
Coagulation factor XII	P00748	-103.4
Complement C1r subcomponent-like protein*	Q9NZP8	-371.6
Complement C1s subcomponent	P09871	-50
Complement factor D	P00746	-4.8
Complement factor I light chain	E7ETH0	-354.5
Dipeptidyl peptidase 2	Q9UHL4	-214.1
Dipeptidyl peptidase 4	P27487	-307.9
Furin	P09958	-113.6
Gamma-glutamyltranspeptidase 1	P19440	-190
Gamma-glutamyltransferase 6	Q6P531	-72
Group XV phospholipase A2	Q8NCC3	-98.4
Haptoglobin	P00738	-373.7
Hepatocyte growth factor activator	Q04756	-138.6
Kallikrein-1 (tissue kallikrein)*	P06870	-476.4
Kallikrein-2	P20151	-27
Kallikrein-11	Q9UBX7	-38.9
Lactotransferrin	P02788	-120.4
Lipoprotein lipase	P06858	-42.9
Lysosomal protective protein (Cathepsin A)*	P10619	-163.9
Lysosomal Pro-X carboxypeptidase	P42785	-186.9
Macrophage stimulating 1	G3XAK1	-82.3
Mannan-binding lectin serine protease 1	P48740	-40.7
Mannan-binding lectin serine protease 2*	O00187	-456.2
Membrane-bound transcription factor site-1 protease	Q14703	-61.2
Myeloblastin (Proteinase 3)*	P24158	-5.9
Neuropathy target esterase	Q8IY17	-13.5
Palmitoyl-protein thioesterase 1	P50897	-53.3
Palmitoyl-protein thioesterase 2	G8JLE1	-81.3
Phosphatidylcholine-sterol acyltransferase	P04180	-174
Plasminogen*	P00747	-556
Platelet-activating factor acetylhydrolase IB subunit alpha	I3L495	-4.5
Platelet-activating factor acethylhydrolase IB subunit beta	P68402	-12
Probable serine carboxypeptidase CPVL	Q9H3G5	-114.8
Prostasin (channel-activating protease 1)	Q16651	-258.5
Prostate specific antigen (Kallikrein-3)*	P07288	-603.5
Prothrombin*	P00734	-841.7
Retinoid-inducible serine carboxypeptidase	Q9HB40	-126.8
Serine protease 23	O95084	-24.3
Serine protease hepsin	P05981	-42.4
Serine protease HTRA1	Q92743	-42.8
Serine protease HTRA3	P83110	-7.1
S-formylgluthathione hydrolase*	P10768	-63.4
Sialate O-acetylesterase*	Q9HAT2	-301.1
Tissue-type plasminogen activator	P00750	-16.9
Transmembrane protease serine 2	O15393	-127.2
Transmembrane protease serine 13	E9PIJ5	-23.2
Tripeptidyl-peptidase 1	O14773	-416.7
Trypsin-1	P07477	-22.5
Tryptase alpha/beta-1	Q15661	-15.8
Urokinase-type plasminogen activator*	P00749	-311
Vitamin K-dependent protein C	P04070	-56.9
Vitamin K-dependent protein Z	P22891	-400.5

^§^Log(e) The base-10 log of the expectation that any particular protein assignment was made at random (*E*-value).

*Serine hydrolase that was identified in an active conformational state with ABPP, affinity purification and MS/MS.

() Alternative nomenclature.

Twelve of the thirteen active serine hydrolases identified with ABPP affinity purification were present on the 2D LC-MS/MS compositional analysis (Table 2). Eleven had high GPM log_10_ (expectation) scores, suggesting that they are high abundance proteins. Notably, myeloblastin (proteinase 3) appeared to be a low abundance protein [log_10_ (-5.9)] and plasma kallikrein was only detected with ABPP affinity purification, which supports the successful isolation of low abundance active proteins. Finally, several high abundance serine hydrolases (vitamin-K dependent protein Z, tripeptidyl-peptidase 1, complement factor I light chain) were not identified with ABPP affinity purification. Taken together, these data suggest that the active serine hydrolases identified were not due to non-specific binding of high abundance enzymes.

**Table 2:** Active serine hydrolases in normal urine (n = 13)

**Serine hydrolase**	**Type**	**Uniprot number**	**MW (kDa)**	**Log(e)**^**§**^	**Potential role(s)**
Kallikrein-1 (tissue kallikrein)	Protease, S1	P06870	28.889	-38.8	1. Cleaves kininogen to kinins, which act on the B1 & B2 receptor.
					2. Luminal TK activates ENaC to increase Na reabsorption.
					3. Luminal TK increases TRPV5 Ca reabsorption via a B2 receptor-dependent mechanism.
					4. Luminal TK inhibits H^+^/K^+^ ATPase to decrease K reasbsorption.
Plasma kallikrein	Protease, S1	P03952	71.370	-5.8	1. Cleaves kininogen to kinins, which act on the B1 & B2 receptor.
Prostate specific antigen (kallikrein-3)	Protease, S1	P07288	28.741	-3.2	1. Liquefaction of semen, for sperm to move freely.
Lysosomal protective protein (cathepsin A)	Protease, S10	P10619	54.466	-57.1	1. Cleaves angiotensin I to angiotensin 1–9, which enhances the kinin effect on the B2 receptor.
Plasminogen^¶^	Protease, S1	P00747	90.569	-3.5	1. Luminal plasmin activates ENaC to increase Na reabsorption.
					2. Luminal plasmin inhibits TRPV5 mediated Ca reabsorption.
					3. Fibrinolysis
Urokinase-type plasminogen activator	Protease, S1	P00749	45.507	-38.2	1. Cleaves the zymogen plasminogen to plasmin.
					2. Fibrinolysis
Prothrombin	Protease, S1	P00734	70.037	-32.4	1. Fibrinolysis
					2. Function in urine unknown
Complement C1r subcomponent-like protein	Protease, S1	Q9NZP8	53.462	-81.7	1. Cleaves pro-C1s, to help activate the classical complement pathway.
Mannan binding lectin serine protease 2	Protease, S1	O00187	20.629	-28.7	1. Cleaves C2 and C4, to help activate the lectin complement pathway.
Myeloblastin (Proteinase 3)	Protease, S1	P24158	27.807	-5.2	1. Neutrophil activation
					2. Cleaves human cathelicidin-18 into antimicrobial peptide LL-37.
Sialate O-acetylesterase	Carboxyl esterase	Q9HAT2	58.315	-48.6	1. Negatively regulates B cell receptor signalling – role in B cell tolerance.
					2. Function in urine unknown
Bile salt-activated lipase	Type B carboxyl esterase/lipase	P19835	79.321	-128.6	1. Lipolysis
					2. Function in urine unknown
S-formylgluthathione hydrolase	Esterase D	P10768	31.462	-6.6	1. Function in urine unknown

*Abbreviations*:*B1* (bradykinin 1 receptor), *B2* (bradykinin 2 receptor), *TK* (tissue kallikrein), *ENaC* (epithelial sodium channel), *TRPV5* (transient receptor potential channel vanilloid subtype 5), *H* (hydrogen), *K* (potassium), *Na* (sodium), *ATP* (adenosine triphosphate).

^§^Log(e) The base-10 log of the expectation that any particular protein assignment was made at random (*E*-value).

^¶^The plasmin peptide identified was: VILGAHQEVN LEPHVQEIEV SR, which is the active serine hydrolase released the plasminogen precursor.

() Alternative nomenclature.

## Discussion

ABPP is a powerful new technique that provides an unbiased, functional analysis of the proteome. Many genomic, transcriptomic and proteomic approaches only identify changes in protein quantity, which does not reflect protein functional status. ABPP offers additional insights over metabolomics, since specific enzymes which are differentially active can be identified for further characterization. This study demonstrates the ease and utility of ABPP with its potential applicability to different renal disease models. This study also provides a unique in-depth activity and compositional analysis of serine hydrolases in normal urine. Serine hydrolase activity frequently, but not invariably, correlates with total protein, thus emphasizing the need for functional proteomic characterization. Interestingly, three active esterases were identified that have limited functional characterization and no previously specified role in the urine, and these may be important targets for further analysis. Equally importantly, we identified active serine proteases involved in key renal physiological processes and innate immunity, and these are discussed below.

Kallikrein-1 (tissue kallikrein) cleaves kininogen to produce vasoactive kinins and their activity is primarily mediated through the bradykinin 1 (B1) and bradykinin (B2) receptors [14]. The pleiotropic effects of kinins include vasodilation, natriuresis, diuresis, anti-fibrotic and anti-hypertrophic actions [15]. Bradykinin-dependent activation of the B2 receptor causes natriuresis by inhibiting sodium reabsorption in the collecting duct [16]. However, tissue kallikrein also acts in a kinin-independent manner to regulate sodium reabsorption [16,17]. Tissue kallikrein is a locally produced regulator that acts luminally on ENaC receptors of the principal cells by cleaving its γ-subunit, thus promoting increased sodium reabsorption [16,17]. While it modulates sodium absorption, its actions are not essential, as tissue kallikrein deficient mice maintain normal blood pressure and extracellular fluid volume status.

Tissue kallikrein is also implicated in renal calcium homeostasis [18]. Calcium is actively reabsorbed in the distal convoluted tubule through the apical transient receptor potential channel vanilloid subtype 5 (TRPV5), transported through the cytosol by calbindin-D_28K_, and then basolaterally transported via the Na^+^/Ca^2+^ exchanger and Ca^2+^ ATPase transporters [19]. Luminal tissue kallikrein stimulates calcium reabsorption by activating the B2 receptor, which results in protein kinase C (PKC)-dependent phosphorylation of TRPV5 [19]. This causes stabilization and accumulation of TRPV5 at the plasma membrane, thereby increasing net calcium reabsorption [19]. Notably, regulation of the TRPV5 receptor was specific to luminal, not basolateral, tissue kallikrein [19].

Tissue kallikrein is also a unique aldosterone-independent kalliuretic factor that allows for rapid adaptation to a dietary potassium load in the cortical collecting duct [20]. Luminal tissue kallikrein promotes potassium secretion by stimulating ENaC activity and sodium reabsorption in the principal cells, as described above [16,20]. Furthermore, tissue kallikrein inhibits potassium reabsorption in intercalated cells by decreasing H^+^/K^+^-ATPase expression and activity, resulting in a net kalliuretic effect [20]. Importantly, luminal tissue kallikrein demonstrated inhibition of H^+^/K^+^-ATPase activity by 70%, whereas basolateral tissue kallikrein had no effect [20].

In the kidney, tissue kallikrein is synthesized primarily in the connecting tubule cells, and to a lesser extent in the distal convoluted tubules and cortical collecting duct [17]. Taken together, these data suggest that tissue kallikrein is synthesized proximally and released into the pro-urine of the tubular lumen to act distally in a paracrine fashion to regulate sodium, calcium and potassium handling. Our observation that tissue kallikrein is present in an active conformational state in normal human urine expands on the mouse and in-vitro work done thus far, and suggests that it may reflect real-time regulation of sodium, calcium and potassium handling. Shedding of active tissue kallikrein in the urine may also represent a rapid means to down-regulate its activity.

Kallikrein-related peptidase (*KLK3*), or prostate specific antigen, is located on the same gene locus as tissue kallikrein (*KLK1*), however its function relates primarily to the liquefaction of semen to allow sperm to move freely, and has no known renal physiological functions. Conversely plasma kallikrein (*KLKB1*) is located on a different gene locus but has very similar physiological functions as tissue kallikrein [21]. Plasma kallikrein cleaves high molecular weight kininogens to release vasoactive kinins that activate the B2 receptor [21]. Plasma kallikrein may potentially contribute to B2-receptor mediated natriuresis and calcium reabsorption, in a manner similar to tissue kallikrein.

Urokinase-type plasminogen activator cleaves the zymogen plasminogen into plasmin, a serine protease. Urinary plasmin directly activates ENaC by cleaving its γ-subunit to promote sodium reabsorption in nephrotic patients and mice [22-24], in a mechanism similar to tissue kallikrein. Svenningsen *et al*. postulated that a defective glomerular filtration barrier allowed for passage of plasmin to activate ENaC, thus contributing to hypertension and edema in nephrotic syndrome [24]. However, the increased sensitivity of ABPP and mass spectrometry techniques utilized in our study permitted the identification of both plasmin and urokinase-type plasminogen activator in healthy individuals. These novel data raise the possibility that urinary plasmin plays a paracrine regulatory role via ENaC in normal renal physiology, not just nephrotic syndrome.

Urinary plasmin from nephrotic individuals also decreases calcium reabsorption by inhibiting the TRPV5 receptor [25]. Urinary plasmin catalyzes protease-activated receptor-1, which promotes phosphorylation of TRPV5 at a different site from tissue kallikrein, resulting in decreased channel pore size and calcium reabsorption [25]. Tudpor *et al*. did not identify plasmin in normal urine by Western blot, but they demonstrated plasmin activity with a plasmin-specific activity assay in normal urine [25], which is consistent with our finding that plasmin is present in an active conformational state in normal urine. Interestingly, Tudpor *et al*. demonstrated that plasmin inhibits calcium influx with 50% inhibitory concentration of ~3nM [25]. Taken together, this suggests that urinary plasmin may play a physiological role at low concentrations, which is readily detectable with mass spectrometry-based techniques.

In a cardiac model, cathespin A localizes to atrial tissue myocytes where it cleaves angiotensin I to release angiotensin 1–9 [26,27]. Angiotensin 1–9 is a bio-active peptide that enhances the kinin effect on the B2 receptor and augments arachidonic acid and nitric oxide release from endothelial cells [26,27]. Grobe *et al*. used MALDI-TOF MS to evaluate bio-active peptides resulting from renal processing of angiotensin II [28], but there is no renal data regarding cathepsin A on angiotensin I. Our observation that active cathepsin A is present in normal urine suggests that it may play a physiological role, such as potentiating the effect of renal-derived bradykinin.

Several of the serine hydrolases detected with ABPP have been shown to be involved in innate immunity. Mannan-binding lectin serine protease 2 (MASP2) participates in activating the lectin pathway of the complement cascade when MASP2 cleaves C4 [29,30]. Complement C1r subcomponent-like protein (C1r-LP), homologous to C1r, participates in activating the classical complement pathway via cleavage of pro-C1s [31]. The role of C1r-LP in complement-mediated functions is still unclear with suggestions of both activating and inhibitory roles [31,32]. Proteinase 3 acts in concert with neutrophil elastase to promote neutrophil activation by cleaving and inactivating the anti-inflammatory progranulin [33]. Furthermore, proteinase 3 has been demonstrated to process human cathelicidin-18 into the antimicrobial peptide LL-37 [34]. Taken together, these observations raise the intriguing possibility that this group of enzymes may play an active role in regulating the innate immunity of the urinary tract.

There are some limitations to this study. First, this is strictly an observational characterization of serine hydrolases in normal individuals. While there is literature to support their activities in urine, these observations are only hypothesis-generating since their physiological role cannot be validated in the current model. Secondly, the affinity-purification step was effective but did not fully retrieve all the FP-TAMRA labelled proteins in the sample. Given the ENaC regulators identified, we anticipated the identification of other ENaC regulators, such as furin and prostasin [23]. Both furin and prostasin were present on the 2D LC-MS/MS compositional analysis, but their active species were not identified with ABPP. One possibility is that tissue kallikrein and plasmin are physiologically dominant ENaC regulators, and furin and prostasin play a limited role if any. However, since all the bands were not enriched with affinity purification we cannot categorically exclude their activity.

The strengths of this study relate to the novel ABPP methodology which provides a functional proteomic characterization that may be more physiologically relevant than a straightforward compositional analysis. Indeed, it provides insight into which enzymes may be active versus filtered peptide fragments that are detectable with highly sensitive MS/MS approaches but may have no function. ABPP allows for simultaneous assessment of activity for most of an enzyme family and this offers clear advantages for the discovery of novel species with as yet undetermined substrate specificities. The ease of visualization and comparison of labeled proteins in different samples offers the potential for rapid comparative analysis of samples. These properties, linked with the potential for affinity purification and MS-based identification of labeled enzymes, markedly enhance the utility of the approach. Critically, identification of specific enzyme activity with ABPP offers the potential for developing rapid colorimetric or fluorometric urine screening assays using immobilized substrates (e.g. urine dipstick).

## Conclusions

In conclusion, this is the first unbiased, functional characterization of the urinary proteome using serine hydrolase ABPP. ABPP identified normal urinary serine hydrolases that are present in an active conformational state. Urinary tissue kallikrein and plasmin were identified and may play a key role in sodium, potassium and calcium homeostasis. Serine proteases involved in complement activation and generation of antimicrobial peptides were also identified which suggests a potential role for regulation of innate immunity of the urinary tract. Novel active esterases with undefined functions were also identified that may be targets for further characterization. Finally, ABPP methodology is a useful tool that could be broadly applied to renal pathophysiological states to identify differentially activated enzymes; develop point-of-care monitoring assays; and potentially identify new therapeutic targets, such as specific enzyme inhibitors.

## Methods

### Activity-based protein profiling

Random, mid-stream urine samples were collected from 18 healthy individuals (male, n = 9 and female, n = 9). Urines were centrifuged at 2000 rpm for 10 minutes at 4°C and the supernatant stored at -80°C for analysis. Urine samples underwent a single freeze/thaw cycle prior to ABPP analysis.

Serine hydrolase ABPP was optimized in normal urines with FP-TAMRA (2 μM) [ActivX FP-TAMRA serine hydrolase probe, Catalogue 88318, Thermo Fisher Scientific USA] at different pH, temperature and reaction times. FP-TAMRA probe incubation was performed at pH 5, 7 and 9 and compared to labeled urines without pH modification; pH 9 was subsequently determined to be optimal (Additional file 2). FP-TAMRA probe incubation was also performed at 20°C, 37°C and 60°C (pH 9), and the weakest FP-TAMRA labeling for males and females was at 20°C (Additional file 3). Therefore, 37°C was chosen as the optimal temperature for ABPP labeling as it is physiological, and we wanted to avoid heat activation of enzymes. Lastly, reaction times were evaluated at pH 9, 37°C for 5, 30 and 90 minutes (Additional file 4) with 90 minutes shown to have optimal FP-TAMRA labeling. Labeled proteins were separated on LDS-PAGE [Rainbow Molecular weight marker RPN 800E, GE] and fluorescence from active enzymes was detected on gel at a wavelength of 534 nm and 20 seconds exposure time [Alpha Innotech – Fluorchem® Q, USA].

Gels were then incubated overnight with SYPRO Ruby ® [Sypro Ruby gel stain, Catalogue S4942, Sigma Aldrich USA] and washed with 7% acetic acid, 10% methanol solution for 20 minutes before scanning at a wavelength of 534 nm [Alpha Innotech – Fluorchem® Q, USA]. Urinary serine hydrolase activity was found to be stable for up to 5 freeze thaw cycles.

### Affinity purification & identification of active serine hydrolases

Normal urine (50 mL) was concentrated to 800 μL using centrifugation (4000 g, 30 minutes, 4°C) with a 30 kDa cut-off [Amicon® UFC903024]. The concentrated sample (200 μl) was labeled with 2 μM FP-TAMRA probe [ActivX FP-TAMRA serine hydrolase probe, Catalogue 88318, Thermo Fisher Scientific USA] at pH 9, 37°C for 90 minutes. Samples were cleaned on Zeba columns [Catalogue# 89891, Pierce] for 2 minutes, 1000 g, at 4°C. Labeled enzymes were co-immunoprecipitated overnight using 10 μl anti-TAMRA antibody [A6397, Invitrogen] bound to 40 μl of Dynabeads® [Dynabeads® protein G, Invitrogen], and then separated with a magnet. For in-gel digestion, tagged proteins were separated on LDS-PAGE, bands excised from gels and analyzed by MS-MS. For in-solution digestion, the Dynabeads® were washed (50 mM PBS, 0.25% Tween 20), and tagged proteins digested with 250 ng trypsin [Promega V5111]. Peptide digests were desalted and purified off-line using ZipTip® C_18_ Pipette Tips [Millipore], were frozen at -80°C and dried using a speed vacuum.

### In-depth compositional analysis of normal urine – 2D MS-MS

Urine protein (32 μg/individual) was pooled from four healthy individuals for analysis. Samples (5 mL) were filtered with a 0.22 μm PVDF syringe filter and then concentrated to ~400 μl with a 3 kDa molecular weight cut-off membrane (Amicon Ultra-15, Millipore) by centrifugation (50 min, 4000 g, 4°C). The concentrated urine was washed 3 times with 4.6 ml ammonium bicarbonate 100 mM. The final volume was adjusted to 500 μl with 100 mM ammonium bicarbonate. The sample was reduced with 12.5 μl 100 mM DTT (30 min, 57°C) and then alkylated with 12.5 μl 500 mM IAA (30 min, room temperature). A final incubation with 20 μl DTT 100 mM was performed (30 min, room temperature) and then trypsin digestion (1/50) was performed overnight (37°C). After digestion, 50 μl of 10% TFA in acetonitrile were added, the sample frozen at -80°C and dried in speed vac. Samples underwent 2D LC/MS-MS for peptide identification.

### Nano RPLC-MS/MS

Samples were analyzed by nano-RPLC-MS/MS using an A splitless Ultra 2D Plus [Eksigent, Dublin, CA] system coupled to a high speed Triple TOF™ 5600 mass spectrometer [AB SCIEX, Concord, Canada]. Peptides were injected via a PepMap100 trap column [0.3 × 5mm, 5 μm, 100 Å, Dionex, Sunnyvale, CA], and a 100 μm × 150mm analytical column packed with 5 μm Luna C18(2) was used prior to MS/MS analysis. Both eluents A (water) and B (98% acetonitrile) contained 0.1% formic acid as an ion-pairing modifier. The tryptic digest was analyzed with 60 minutes gradient. Eluent B had a gradient from 0% to 35% over 48 minutes, 35% to 85% in 1 minute and was kept at 85% for 5 minutes at a flow rate of 500 nL/min. Key parameter settings for the TripleTOF 5600 mass spectrometer were as follows: ionspray voltage floating (ISVF) 3000 V, curtain gas (CUR) 25, interface heater temperature (IHT) 150, ion source gas 1 (GS1) 25, declustering potential (DP) 80 V. All data was acquired using information-dependent acquisition (IDA) mode with Analyst TF 1.5 software [AB SCIEX, USA]. For IDA parameters, 0.25 s MS survey scan in the mass range of 400–1250 were followed by 20 MS/MS scans of 100 ms in the mass range of 100–1600 (total cycle time: 2.3 s). Switching criteria were set to ions greater than mass to charge ratio (m/z) 400 and smaller than m/z 1250 with a charge state of 2–5 and an abundance threshold of more than 150 counts. Former target ions were excluded for 5 seconds. A sweeping collision energy setting of 37 ± 15 eV was applied to all precursor ions for collision-induced dissociation.

### Database analysis and protein identification

Spectra files were generated using Analyst® TF 1.5.1 Software and converted into mascot generic file format (.mgf) using AB SCIEX MS Data converter [AB SCIEX, Foster City, CA]. These files containing the MS/MS spectra information were submitted for protein identification by the X!Tandem GPM (http://www.thegpm.org). The following parameters were used: (i) enzyme, trypsin; (ii) one missed cleavage allowed; (iii) fixed modification, carbamidomethylation of cysteines (for in gel only); (iv) variable modification, oxidation of methionine; (v) peptide tolerance, 3.0 Da; and (vi) MS/MS tolerance, 0.4 Da. The 2D LC-MS/MS data of normal urinary proteins (n = 1846) were annotated against a list of serine hydrolases (n = 244) described by Cravatt *et al.*[8] and 4 other serine hydrolases identified in Uniprot. Serine hydrolases identified with high confidence GPM log_10_ (expectation) scores of less than -3 (n = 62) derived from this annotated list were used to generate Table 1.

## Abbreviations

2D LC-MS/MS: Two-dimensional liquid chromatography tandem mass spectrometry; ABPP: Activity-based protein profiling; B1: Bradykinin 1 receptor; B2: Bradykinin 2 receptor; C1r-LP: Complement C1r subcomponent-like protein; Ca+: Calcium; ENaC: Epithelial sodium channel; FP-TAMRA: Fluorophosphonate probe tetramethylrhodamine; GPM: Global Proteome Machine; H+: Hydrogen; K+: Potassium; MALDI-TOF MS: Matrix-assisted laser desorption/ionization time-of-flight mass spectrometry; MASP2: Mannan-binding lectin serine protease 2; Na+: Sodium; PKC: Protein kinase C; SDS-PAGE: Sodium dodecyl sulphate polyacrylamide gel electrophoresis; TRPV5: Transient receptor potential channel vanilloid subtype 5.

## Competing interests

The authors declare that they have no competing interests.

## Authors’ contributions

MN conducted the ABPP experiments and contributed to writing the manuscript. JH and JAW contributed to the experimental design, data analysis and writing of the manuscript. OK and PE conducted the MS-MS work. CR, MR, DR and PN contributed to revising the manuscript for important intellectual content. All authors read and approved the final manuscript.

## Supplementary Material

Additional file 1 **Specificity of Affinity purification of FP-TAMRA labelled urine.** Beads conjugated with an anti-HIV antibody did not enrich FP-TAMRA labelled bands. 1) FP-TAMRA labelled starting material. 2) Material eluted from anti-HIV column. 3) Starting material total protein. 4) Protein eluted from anti-HIV column. Click here for file

Additional file 2 Activity-based protein profiling (ABPP) of male (A) and female (B) urines without pH modification and at pH 5, 7 and 9. Click here for file

Additional file 3 Activity-based protein profiling (ABPP) of normal male (A) and female (B) urines at different temperatures, pH 9. Click here for file

Additional file 4 Activity-based protein profiling (ABPP) of normal male and female urines with different incubation times (5 min, 30 min, 90 min), 37°C, pH 9. Click here for file
