# Supplementary material for: Proteomic characterization of serine hydrolase activity and composition in normal urine
Source: Clin Proteomics. 2013 Nov 15;10(1):17. doi: 10.1186/1559-0275-10-17 (PMC4225696; doi:10.1186/1559-0275-10-17)
Supplement: Additional file 1 — Specificity of Affinity purification of FP-TAMRA labelled urine. Beads conjugated with an anti-HIV antibody did not enrich FP-TAMRA labelled bands. 1) FP-TAMRA labelled starting material. 2) Material eluted from anti-HIV column. 3) Starting material total protein. 4) Protein eluted from anti-HIV column. [file 1559-0275-10-17-S1.ppt]

## Slide 1
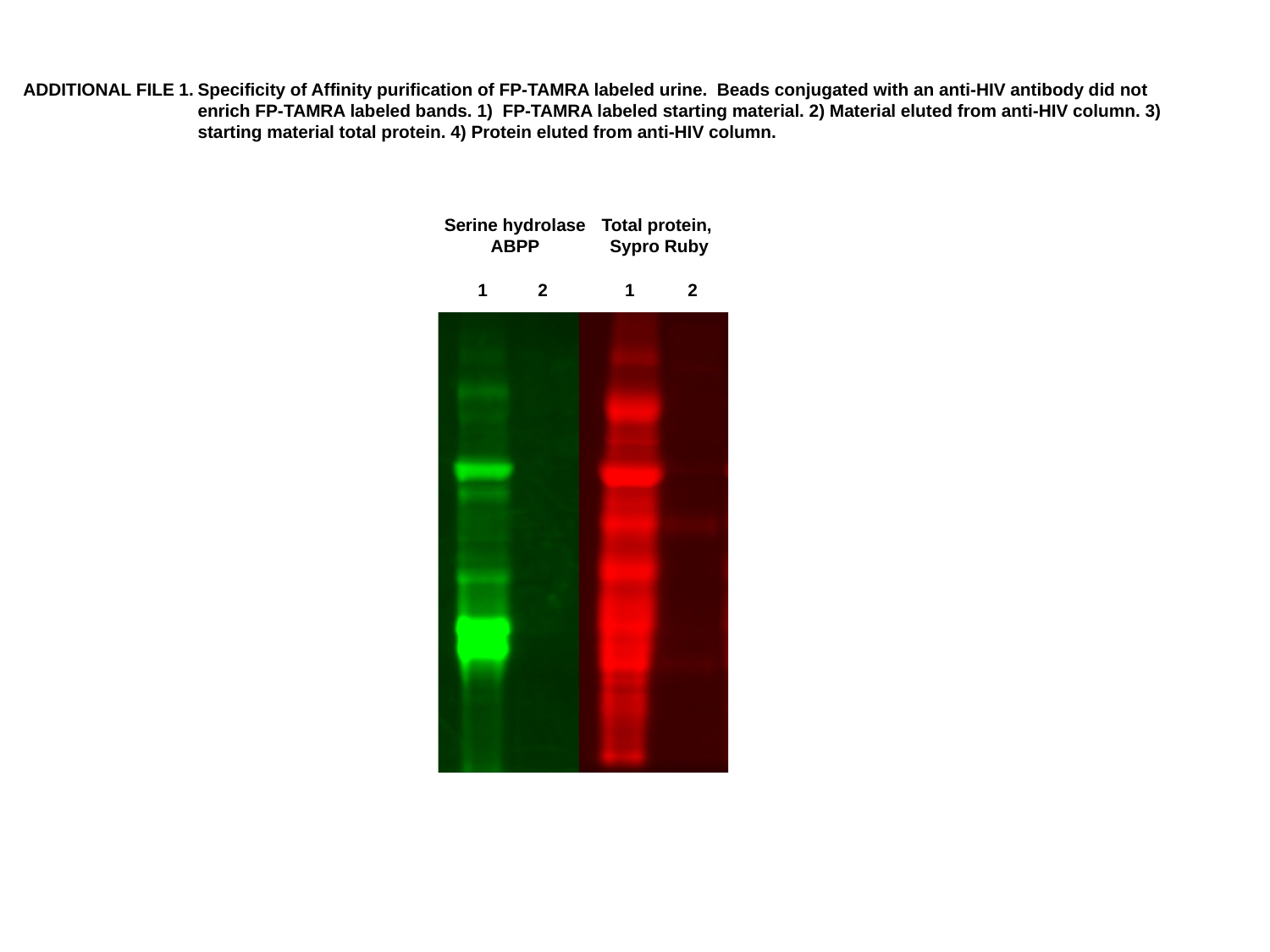

ADDITIONAL FILE 1.	Specificity of Affinity purification of FP-TAMRA labeled urine. Beads conjugated with an anti-HIV antibody did not 		enrich FP-TAMRA labeled bands. 1) FP-TAMRA labeled starting material. 2) Material eluted from anti-HIV column. 3) 		starting material total protein. 4) Protein eluted from anti-HIV column.
Serine hydrolase
ABPP
Total protein,
Sypro Ruby
1
2
1
2
