# Supplementary material for: Proteomic characterization of serine hydrolase activity and composition in normal urine
Source: Clin Proteomics. 2013 Nov 15;10(1):17. doi: 10.1186/1559-0275-10-17 (PMC4225696; doi:10.1186/1559-0275-10-17)
Supplement: Additional file 2 — Activity-based protein profiling (ABPP) of male (A) and female (B) urines without pH modification and at pH 5, 7 and 9. [file 1559-0275-10-17-S2.ppt]

## Slide 1
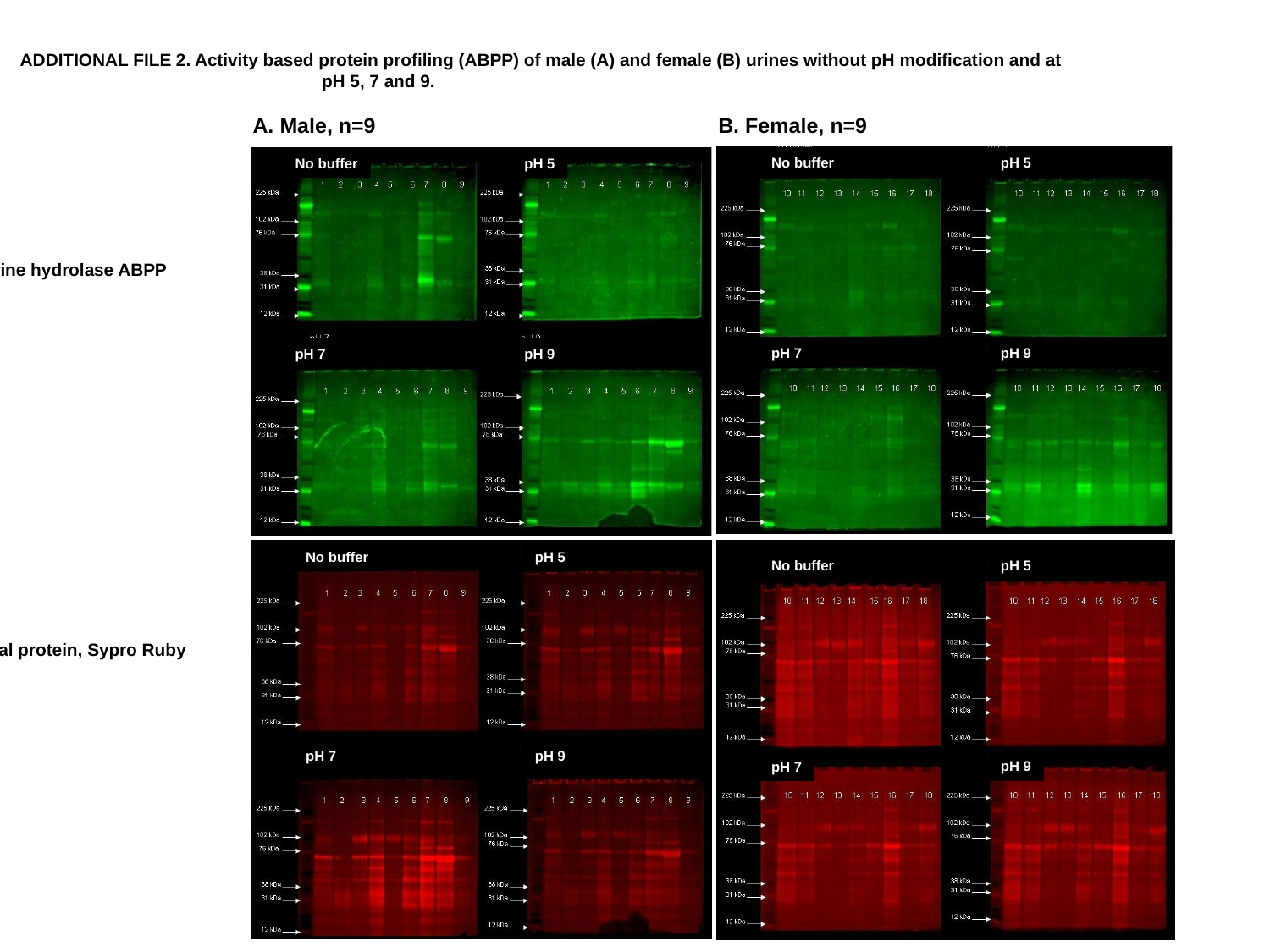

ADDITIONAL FILE 2.	Activity based protein profiling (ABPP) of male (A) and female (B) urines without pH modification and at
			pH 5, 7 and 9.
A. Male, n=9
B. Female, n=9
No buffer
pH 5
No buffer
pH 5
Serine hydrolase ABPP
pH 9
pH 7
pH 9
pH 7
No buffer
pH 5
No buffer
pH 5
Total protein, Sypro Ruby
pH 9
pH 7
pH 9
pH 7
