# Supplementary material for: Proteomic characterization of serine hydrolase activity and composition in normal urine
Source: Clin Proteomics. 2013 Nov 15;10(1):17. doi: 10.1186/1559-0275-10-17 (PMC4225696; doi:10.1186/1559-0275-10-17)
Supplement: Additional file 3 — Activity-based protein profiling (ABPP) of normal male (A) and female (B) urines at different temperatures, pH 9. [file 1559-0275-10-17-S3.ppt]

## Slide 1
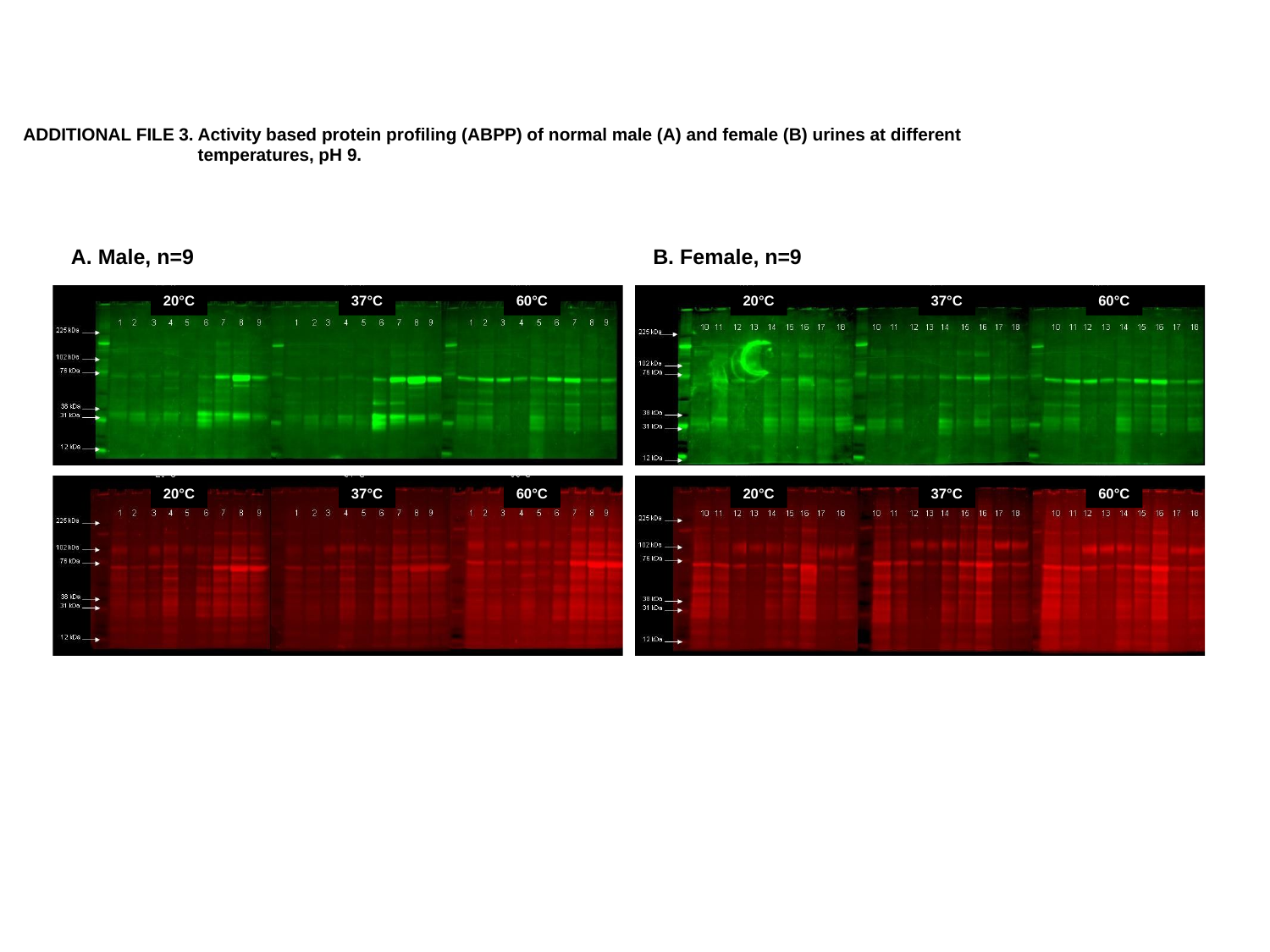

ADDITIONAL FILE 3.	Activity based protein profiling (ABPP) of normal male (A) and female (B) urines at different 			temperatures, pH 9.
A. Male, n=9
B. Female, n=9
20°C
37°C
60°C
20°C
37°C
60°C
20°C
37°C
60°C
20°C
37°C
60°C
