# Supplementary material for: Proteomic characterization of serine hydrolase activity and composition in normal urine
Source: Clin Proteomics. 2013 Nov 15;10(1):17. doi: 10.1186/1559-0275-10-17 (PMC4225696; doi:10.1186/1559-0275-10-17)
Supplement: Additional file 4 — Activity-based protein profiling (ABPP) of normal male and female urines with different incubation times (5 min, 30 min, 90 min), 37°C, pH 9. [file 1559-0275-10-17-S4.ppt]

## Slide 1
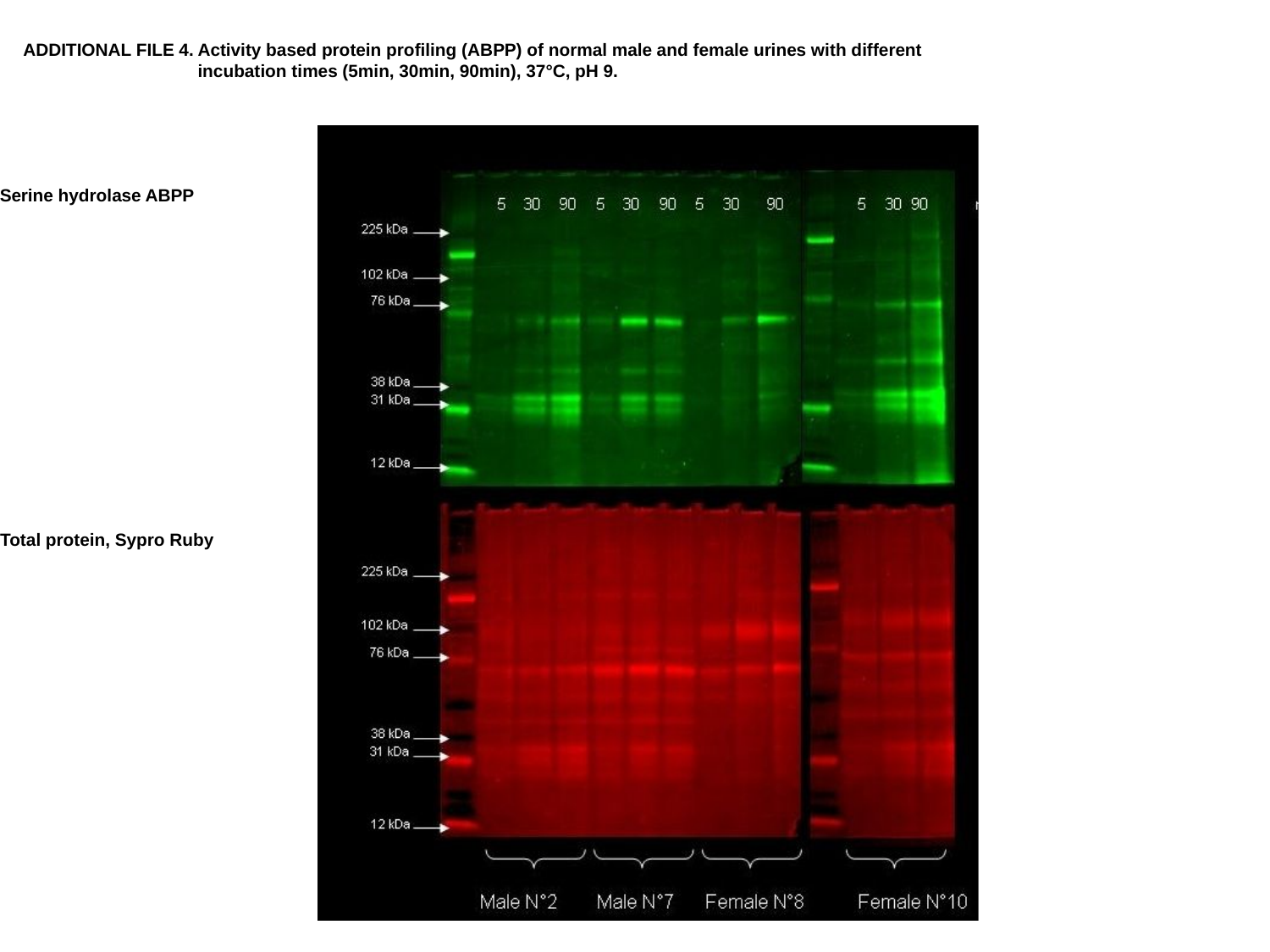

ADDITIONAL FILE 4.	Activity based protein profiling (ABPP) of normal male and female urines with different 				incubation times (5min, 30min, 90min), 37°C, pH 9.
Serine hydrolase ABPP
Total protein, Sypro Ruby
